# Supplementary material for: Machine Learning Accelerates Crystallization for Structure Determination
Source: Angew Chem Int Ed Engl. 2026 May 4;65(25):e1218503. doi: 10.1002/anie.1218503 (PMC13266958; doi:10.1002/anie.1218503)

checkCIF/PLATON report

Structure factors have been supplied for datablock(s) lcz093

THIS REPORT IS FOR GUIDANCE ONLY. IF USED AS PART OF A REVIEW PROCEDURE FOR PUBLICATION, IT SHOULD NOT REPLACE THE EXPERTISE OF AN EXPERIENCED CRYSTALLOGRAPHIC REFEREE.

No syntax errors found. CIF dictionary Interpreting this report

Datablock: lcz093

Bond precision:	C-C = 0.0115 A		Wavelength=1.54184
Cell:	a=12.8384(2)	b=20.2216(3)	c=13.6185(3)
	alpha=90	beta=107.272(2)	gamma=90
Temperature:	100 K		

	Calculated	Reported
Volume	3376.11(11)	3376.11(11)
Space group	P 21/c	P 1 21/c 1
Hall group	-P 2ybc	-P 2ybc
Moiety formula	C25 H15 Ag3 F18 N6 O2 S	C15 H3 Ag3 F18 N6, C10 H12 O2 S
Sum formula	C25 H15 Ag3 F18 N6 O2 S	C25 H15 Ag3 F18 N6 O2 S
Mr	1129.10	1129.10
Dx,g cm-3	2.221	2.221
Z	4	4
Mu (mm-1)	15.691	15.691
F000	2168.0	2168.0
F000'	2179.61	
h,k,lmax	16,25,17	16,25,17
Nref	7203	6838
Tmin,Tmax	0.113,0.095	0.104,1.000
Tmin'	0.009	

Correction method= # Reported T Limits: Tmin=0.104 Tmax=1.000
AbsCorr = MULTI-SCAN

```
Data completeness= 0.949            Theta(max)= 77.799

                                                   wR2(reflections)=
R(reflections)= 0.0587( 6086)                      0.1596( 6838)

S = 1.081                Npar= 498
```

The following ALERTS were generated. Each ALERT has the format
test-name_ALERT_alert-type_alert-level.
Click on the hyperlinks for more details of the test.

Alert level C

```
PLAT042_ALERT_1_C Calc. and Reported MoietyFormula Strings  Differ     Please Check
              Calc: C25 H15 Ag3 F18 N6 O2 S
              Rep.: C15 H3 Ag3 F18 N6, C10 H12 O2 S
PLAT213_ALERT_2_C Atom F00S            has ADP max/min Ratio .....         3.5 prolat
PLAT213_ALERT_2_C Atom F01G            has ADP max/min Ratio .....         3.5 prolat
PLAT213_ALERT_2_C Atom F01H            has ADP max/min Ratio .....         3.7 prolat
PLAT220_ALERT_2_C NonSolvent   Resd 1  F   Ueq(max)/Ueq(min) Range         4.0 Ratio
PLAT234_ALERT_4_C Large Hirshfeld Difference F01G     --C01F     .        0.16 Ang.
PLAT342_ALERT_3_C Low Bond Precision on  C-C Bonds ..............     0.01155 Ang.
PLAT906_ALERT_3_C Large K Value in the Analysis of Variance ......       7.285 Check
PLAT906_ALERT_3_C Large K Value in the Analysis of Variance ......       2.254 Check
PLAT911_ALERT_3_C Missing FCF Refl Between Thmin & STh/L=    0.600          48 Report
                1  1  0,   0  2  0,   4 16  1,   0  0  2,  13  7  2,  -4 23  4,
              -12 14  5,  10  4  6,   7 17  6,   8  6  7,   9  6  7,   6 17  7,
                8  7  8,   5  9  8,   7  9  9,   8  9  9,   1 12  9,   7  9 10,
                7 10 10,   3 11 10,   3 12 10,   0 13 10,   1 13 11,   2 13 11,
                4 13 11,  -1  0 12,   2  1 12,   0 15 12,  -1 16 12,   1  1 13,
                  (   18 More Missing: see the .ckf listing file)
PLAT934_ALERT_3_C Number of (Iobs-Icalc)/Sigma(W) > 10 Outliers ..           1 Check
               -6  9 16,
PLAT972_ALERT_2_C Check Calcd Resid. Dens.  0.75Ang From Ag03            -1.54 eA-3
PLAT977_ALERT_2_C Check Negative Difference Density on H00V      .       -0.32 eA-3
```

Alert level G

```
PLAT003_ALERT_2_G Number of Uiso or U(i,j)  Restrained non-H-Atoms           4 Report
PLAT083_ALERT_2_G SHELXL Second Parameter in WGHT  Unusually Large       33.59 Why ?
PLAT186_ALERT_4_G The CIF-Embedded .res File Contains ISOR Records           2 Report
PLAT242_ALERT_2_G Low    'MainMol' Ueq as Compared to Neighbors of        C010 Check
PLAT242_ALERT_2_G Low    'MainMol' Ueq as Compared to Neighbors of        C013 Check
PLAT242_ALERT_2_G Low    'MainMol' Ueq as Compared to Neighbors of        C014 Check
PLAT242_ALERT_2_G Low    'MainMol' Ueq as Compared to Neighbors of        C01A Check
PLAT242_ALERT_2_G Low    'MainMol' Ueq as Compared to Neighbors of        C01F Check
PLAT242_ALERT_2_G Low    'MainMol' Ueq as Compared to Neighbors of        C01I Check
PLAT434_ALERT_2_G Short Inter HL..HL Contact F009     ..F01H     .        2.78 Ang.
                                          1-x,1/2+y,3/2-z  =      2_656 Check
PLAT720_ALERT_4_G Number of Unusual/Non-Standard Labels .........          70 Note
              Ag01    Ag02    Ag03    S004    F005    F006    F007    F008
              F009    O00A    F00B    O00C    F00D    F00E    F00F    F00G
              F00H    N00I    N00J    N00K    F00L    F00M    N00N    N00O
              N00P    F00Q    C00R    F00S    F00T    C00U    C00V    H00V
              C00W    C00X    H00X    C00Y    H00Y    C00Z    C010    C011
```

```
              H011    C012    C013    C014    C015    C016    H016    C017
              H017    C018    H018    C019    H019    C01A    C01B    C01C
              C01D    H01A    H01B    H01C    C01E    H01E    C01F    F01G
              F01H    C01I    C01J    H01D    H01F    H01G
PLAT860_ALERT_3_G Number of Least-Squares Restraints ............         24 Note
PLAT883_ALERT_1_G Absent Datum for _atom_sites_solution_primary ..     Please Do !
PLAT912_ALERT_4_G Missing # of FCF Reflections Above STh/L=  0.600        317 Note
PLAT933_ALERT_2_G Number of HKL-OMIT Records in Embedded .res File         35 Note
                1  4 14,   0  5 15,   0  6 15,   0  4 14,  -1  7 15,   0  7 15,
               -2  3 15,  -2  1 15,  -3  7 16,  -2  4 16,  -1 10 15,  -2  6 16,
               -1  1 14,   2 13 11,  -4  8 16,  -2  5 16,  -3  9 16,  -2  7 16,
               -2 11 15,   3 12 10,  -1  4 14,  -5  9 16,   1  1 13,  -2  8 16,
               -5  7 16,   5  9  8,   1 13 11,  10  4  6,  -3 12 15,  -3  8 16,
               -3  5 15,   2  1 12,   3 11 10,  -1 16 12,  -3 11 15,
PLAT941_ALERT_3_G Average HKL Measurement Multiplicity ..........        2.4 Low
PLAT969_ALERT_5_G The 'Henn et al.' R-Factor-gap value ..........      3.909 Note
              Predicted wR2: Based on SigI**2  4.08 or SHELX Weight 14.76
PLAT978_ALERT_2_G Number C-C Bonds with Positive Residual Density.          0 Info
```

---

0 **ALERT level A** = Most likely a serious problem - resolve or explain
0 **ALERT level B** = A potentially serious problem, consider carefully
13 **ALERT level C** = Check. Ensure it is not caused by an omission or oversight
18 **ALERT level G** = General information/check it is not something unexpected

2 ALERT type 1 CIF construction/syntax error, inconsistent or missing data
17 ALERT type 2 Indicator that the structure model may be wrong or deficient
7 ALERT type 3 Indicator that the structure quality may be low
4 ALERT type 4 Improvement, methodology, query or suggestion
1 ALERT type 5 Informative message, check

Validation response form

Please find below a validation response form (VRF) that can be filled in and pasted into your CIF.

```
# start Validation Reply Form
_vrf_PLAT042_lcz093
;
PROBLEM: Calc. and Reported MoietyFormula Strings  Differ     Please Check
RESPONSE: ...
;
_vrf_PLAT213_lcz093
;
PROBLEM: Atom F00S            has ADP max/min Ratio .....        3.5 prolat
RESPONSE: ...
;
_vrf_PLAT220_lcz093
;
PROBLEM: NonSolvent   Resd 1  F   Ueq(max)/Ueq(min) Range        4.0 Ratio
RESPONSE: ...
;
_vrf_PLAT234_lcz093
;
PROBLEM: Large Hirshfeld Difference F01G     --C01F     .       0.16 Ang.
RESPONSE: ...
;
```

```
_vrf_PLAT342_lcz093
;
PROBLEM: Low Bond Precision on  C-C Bonds ...............    0.01155 Ang.
RESPONSE: ...
;
_vrf_PLAT906_lcz093
;
PROBLEM: Large K Value in the Analysis of Variance ......      7.285 Check
RESPONSE: ...
;
_vrf_PLAT911_lcz093
;
PROBLEM: Missing FCF Refl Between Thmin & STh/L=    0.600         48 Report
RESPONSE: ...
;
_vrf_PLAT934_lcz093
;
PROBLEM: Number of (Iobs-Icalc)/Sigma(W) > 10 Outliers ..          1 Check
RESPONSE: ...
;
_vrf_PLAT972_lcz093
;
PROBLEM: Check Calcd Resid. Dens.  0.75Ang From Ag03           -1.54 eA-3
RESPONSE: ...
;
_vrf_PLAT977_lcz093
;
PROBLEM: Check Negative Difference Density on H00V      .      -0.32 eA-3
RESPONSE: ...
;
# end Validation Reply Form
```

It is advisable to attempt to resolve as many as possible of the alerts in all categories. Often the minor alerts point to easily fixed oversights, errors and omissions in your CIF or refinement strategy, so attention to these fine details can be worthwhile. It is up to the individual to critically assess their own results and, if necessary, seek expert advice.

PLATON version of 17/09/2025; check.def file version of 17/09/2025

---

duplicate check

No duplication found

Datablock lcz093 - ellipsoid plot

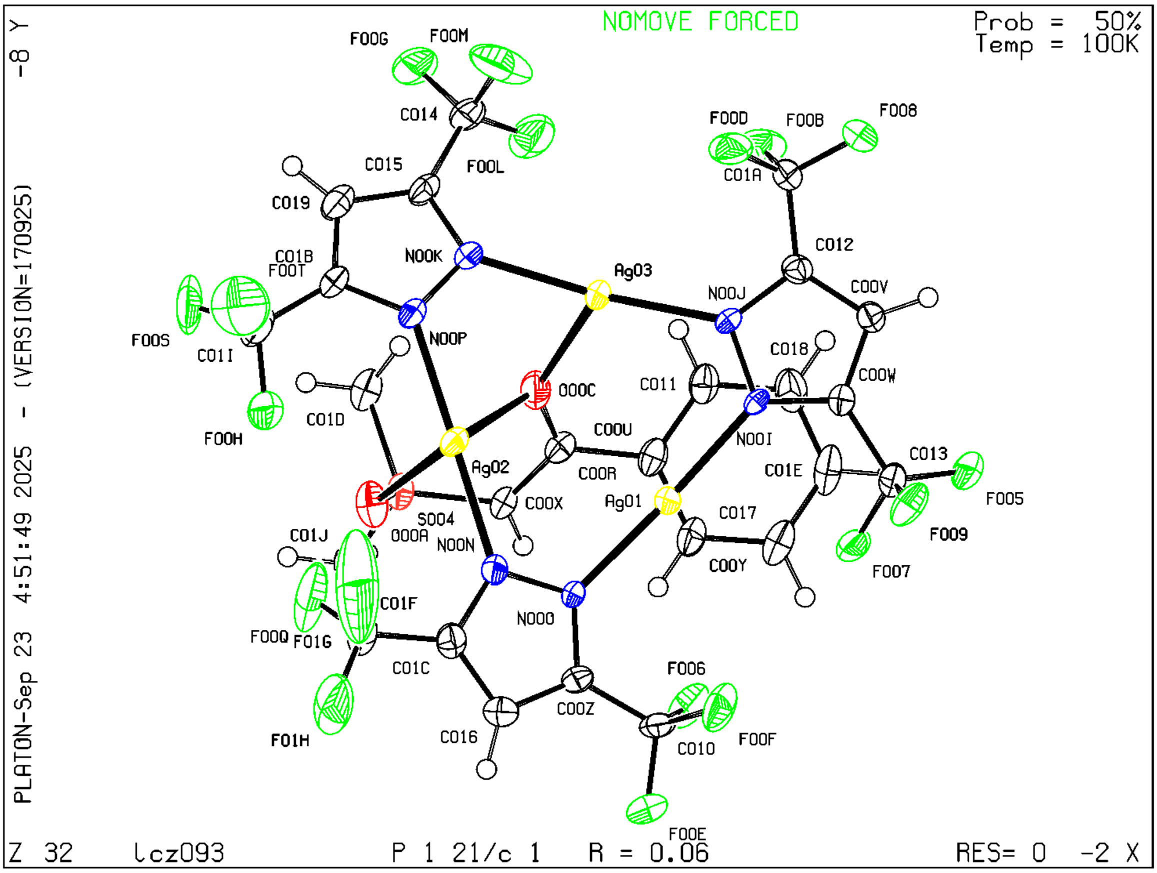

Supplement: Supplementary file 2 — Supporting File 2: anie72462‐sup‐0002‐DataFile.zip. [file ANIE-65-e1218503-s002.zip › supplementary_materials_crystallographic_data submit.zip!/cif_and_checkcif(1-114)/Ag3Pz3_93/Ag3Pz3_93_checkcif.pdf]
